# Prevalence of Quinolone Resistance Among Extended-Spectrum β -Lactamase Producing Uropathogenic *Klebsiella pneumoniae*

**DOI:** 10.5812/jjm.10887

**Published:** 2014-06-01

**Authors:** Fereshteh Raei, Fereshteh Eftekhar, Mohammad Mehdi Feizabadi

**Affiliations:** 1 Department of Microbiology, Faculty of Biological Sciences, Shahid Beheshti University, General Campus, Tehran, IR Iran; 2 Department of Microbiology, School of Medicine, Tehran University of Medical Sciences, Tehran, IR Iran

**Keywords:** *Klebsiella pneumonia*e, quinolone, β -Lactamase

## Abstract

**Background:**

Extended-spectrum β-lactamase (ESBL) production is the major resistance mechanism to β-lactam antibiotics in *Enterobacteriaceae*. In addition, emergence of plasmid-mediated quinolone resistance (PMQR) in ESBL-producing isolates has become a global threat for treatment of these infections.

**Objectives:**

We investigated the association between ESBL production and quinolone resistance in urinary isolates of *K. pneumoniae*.

**Patients and Methods:**

A total of 196 urinary isolates of *K. pneumoniae *were collected from Imam Hussein Hospital in Tehran during a four year period (2008-2012). Antibiotic susceptibility was determined by disc diffusion and ESBL production was screened using the phenotypic confirmatory test (PCT).

**Results:**

All isolates were susceptible to imipenem. Resistance to piperacillin and cefotaxime were 66.3% and 50.5%, respectively. Resistance to ceftazidime, amoxiclave, aztreonam, ceftriaxone, cefepime, nitrofurantoin, gentamicin, ciprofloxacin, nalidixic acid, ofloxacin, norfloxacin, levofloxacin, amikacin and pipracilin/tazobactam were less than 50%. ESBL production was detected in 92 isolates (46.9%) of which, 61.9% were resistant to nalidixic acid and 65.2% to ciprofloxacin. Multidrug-resistance was observed in 96.7% of ESBL producers.

**Conclusions:**

Our results showed coexistence of ESBL and quinolone resistance in the majority of the uropathogenic *K. pneumoniae *test isolates suggesting that care should be taken for the choice of antibiotic therapy.

## 1. Background

*Klebsiella pneumoniae* is an important cause of urinary tract infections (UTIs), pneumonia and intra-abdominal infections in hospitalized immune compromised patients with severe underlying diseases (1). β-lactam antibiotics are commonly used for treatment of *Enterobacteriaceae* related infections. However, resistance to these agent shas increased worldwide mostly due to β-lactamasesproduction (2). Among these enzymes, extended spectrum β-lactamases (ESBLs) have increased in response to the extensive use of extended-spectrum β-lactam antibiotics (3, 4). In addition, ESBL producing bacteria are typically associated with multidrug resistance since multiple resistance genes often reside on the same plasmid. Coexistence of ESBL production with resistance to quinolones, aminoglycosides and trimethoprim–sulfamethoxazole has been shown in a Korean study (4). 

Quinolones are broad-spectrum antibacterial agents, commonly used for treatment of infections both in human and veterinary medicine. As a result, enhanced level of quinolone resistance has occurred in recent years (5). For example, fluoroquinolones such as ciprofloxacin, previously shown to have excellent activity against clinical isolates of *Klebsiella*, have become less effective due to their extensive use (5, 6). Early studies have shown that quinolone resistance arises by mutations in topoisomerase subunits as well as changes in the expression of efflux pumps and porins that control the accumulation of these agents inside the bacterial cell (7). 

The discovery of plasmid-mediated quinolone resistance (PMQR) in the late 1990s added a new dimension to quinolone resistance (8, 9). Coproduction of ESBLs and PMQR proteins can be a major concern. There are no reports on the prevalence of PMQR or its correlation with ESBL production in *K. pneumoniae *isolates in Iran. However, one study reported the coexistence of PMQR and ESBL genes in *Escherichia coli* (10).

## 2. Objectives

The aim of this study was to determine the prevalence of quinolone resistance in relation to ESBL production in clinical urinary isolates of *K. pneumonia*e, collected from Imam Hussein Hospital in Tehran during a four year period (2008-2012).

## 3. Patients and Methods

### 3.1. Bacterial Strains

Three sets of bacteria were isolated from patients admitted to Imam Hussein Hospital in Tehran: March to August 2008 (38 isolates), July 2010 to January 2011 (52 isolates) and February to October 2012 (106 isolates). All isolates (n = 196) were identified by their characteristic appearance and standard biochemical tests (11). *K. pneumoniae* strain ATCC 10031 was used as the antibiotic susceptible control. *K. pneumoniae *207L and 550L (accession numbers: GQ470427 and GQ470460) were kindly provided by Dr. Feizabadi and were ESBL positive controls.

### 3.2. Antimicrobial Susceptibility Testing

Susceptibility to antimicrobial agents was determined as recommended by Clinical and Laboratory Standards Institute (CLSI, 2011) using commercially available discs (Mast, UK) including: imipenem (10 μg), gentamicin (10 μg), amikacin (30 μg), ciprofloxacin (5 μg), nalidixic acid (30 μg), norfloxacin (10 μg), levofloxacin (5 μg), ofloxacin (5 μg), nitrofurantoin (100 μg), piperacillin-tazobactam (100/10 μg), cefotaxime (30 μg), ceftazidime (30 μg), ceftriaxone (30 μg), cefepieme (30 μg), aztreonam (30 μg), nitrofurantoin (300 μg) and amoxicillin-clavulanic acid (20/10 μg) (12).

### 3.3. Initial Screening for ESBL Production

The initial *in vitro* susceptibility testing was performed using the CLSI procedure with ceftazidime (30 μg), cefotaxime (30 μg), ceftriaxone (30 μg), aztreonam (30 μg) and cefpodoxime (Mast, UK, 30 μg). As recommended by the CLSI, zone diameters of ≤ 27 mm for aztreonam and cefotaxime, ≤ 25 mm for ceftriaxone, ≤ 22 mm for ceftazidime and ≤ 17 mm for cefpodoxime indicated probable ESBL production and the isolates were further studied by the phenotypic confirmatory test (12).

### 3.4. Phenotypic Confirmatory Test for ESBL Production

Ceftazidime (30 μg) and cefotaxime (30 μg) alone or in combination with clavulanic acid (10 μg) (Mast, UK), were placed on Muller Hinton agar (Lioflichem, Italy) plates previously inoculated with the test organism and were incubated at 37**°**C for 16-18 hours. An increase of > 5 mm in the zone diameter of the antibiotic in combination with clavulanic acid compared to the antibiotic alone was recorded as an indication of ESBL production (12). To compare the antibiotic resistance profiles of ESBL and non-ESBL producing isolates, non-parametric analysis using the two-tailed Mann-Whitney U test was performed allowing for continuous variables, independent groups and non-normal distribution in SPSS 20 (SPSS, Inc, Chicago, IL, USA). Bivariate Spearman's rank correlation test was used to determine the association between resistance to nalidixic acid and ciprofloxacin in ESBL producers.

## 4. Results

**Figure 1.**
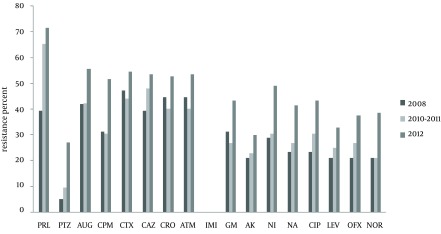
Comparison of Antibiotic Resistance Rates of *K. pneumoniae* in Three Time Periods PRL: piperacillin; PTZ: piperacillin/tazobactam; AUG: amoxiclav; NF: nitrofurantoin; GM: gentamicin; AK: amikacin; CAZ: ceftazidime; CRO: cefriaxone; CTX: cefotaxime; ATM: aztreonam; IMP: imipenem; NA: nalidixic acid; CIP: ciprofloxacin; LEV: levofloxacin; OFX: ofloxacin; NOR: norfloxacin

The antibiotic resistance rates among the urinary isolates of *K. pneumoniae *were as follows; piperacillin 66.3%, cefotaxime 50.5%, ceftazidime and amoxiclav 49.4%, aztreonam 48.4%, ceftriaxone 47.9%, cefepime 42.3%, nitrofurantoin 40.3%, gentamicin 36.7%, ciprofloxacin 36.2%, nalidixic acid 34.1%, ofloxacin 31.6%, norfloxacin 30.6%, levofloxacin 28.5%, amikacin 22.4% and pipracillin-tazobactam 18.3%. All isolates were susceptible to imipenem (Figure 1). Intermediate resistance rates to all antibiotics were below 10% and were not included in Figure 1. 

The only exception was observed for piperacillin/tazobactam where 20.4% of the isolates showed intermediate resistance compared to 9.6% found for piperacillin alone. Figure 1 also shows that antibiotic resistance rates generally increased over time and the last set of isolates collected in 2012 were more resistant to all antibiotics. The highest rates of resistance were observed towards β-lactam antibiotics (ceftriaxone, cefotaxime, pipracillin, ceftazidime, cefepime) and aztreonam. The low rate of resistance to piperacillin/tazobactam also confirms β-lactamase production by the isolates. The phenotypic confirmatory test (PCT) results showed that 92 (46.9%) isolates were ESBL producers of which, 15 belonged to the 2008 set (39.47%), 22 to the 2010-2011 set (42.31%) and 55 to the 2012 collection (51.89%). Table 1 shows that all ESBL positive isolates were significantly more resistant to the tested antibiotics compared to the isolates that did not produce ESBL, except for amoxiclav and imipenem (P < 0.05). In fact, multidrug-resistance (resistance to at least three classes of antibiotics) was observed in 96.7% of ESBL producers compared to 17.3% in non-producers. Among the quinolones, resistance to nalidixic acid and ciprofloxacin were significantly correlated in ESBL producers (Spearman's rank correlation test; r = 0.903, P < 0.05).

**Table 1.** Comparison of Antibiotic Resistance Between non-ESBL and ESBL Producing *K. pneumoniae *Urinary Isolates^a^

Antibiotic	Resistance (%)		
	ESBL Isolates	Non-ESBL Isolates	P Value^b^
**Cefepime**	86.9	2.8	< 0.05
**Cefotaxime**	95.6	10.5	< 0.05
**Ceftriaxone**	96.7	4.8	< 0.05
**Ceftazidime**	89.1	14.4	< 0.05
**Piperacillin**	93.4	7.5	< 0.05
**Amoxicillin/clavulanate**	48.9	50	NS
**Piperacillin/tazobactam**	32.6	5.7	< 0.05
**Aztreonam**	93.4	8.6	0.05
**Imipenem**	0.00	0.00	NS
**Gentamicin**	75.0	2.8	< 0.05
**Amikacin**	52.1	3.8	< 0.05
**Nalidixic acid**	61.9	9.6	< 0.05
**Ciprofloxacin**	65.2	10.5	< 0.05
**Levofloxacin**	52.1	7.6	< 0.05
**Ofloxacin**	56.5	9.6	< 0.05
**Norfloxacin**	58.6	5.7	< 0.05
**Trimethoprim**	77.1	17.3	< 0.05
**Nitrofurantoin**	54.3	27.8	< 0.05

^a^ Abbreviations: NS, not significant

^b^ P < 0.05 values were determined using the two-tailed Mann-Whitney test showing significance at the 95% confidence interval.

## 5. Discussion

*K. pneumoniae *is the second cause of urinary tract infections (7%) in Iran, next to *E. coli* (13). Beta-lactamase production, specifically the ESBL type β-lactamases, limits the use of β-lactam antibiotics as effective therapeutic agents. As shown in this study, piperacillin-tazobactam (18.3%) was significantly lower compared to piperacillin (66.3%). However, up to 20.4% of the isolates showed intermediate resistance to this combination, suggesting that its use should be limited to the susceptible isolates. ESBL-producing isolates of this study showed high multidrug-resistance. 

The clinical relevance of multidrug resistance among ESBL producing *Enterobacteriaceae* is of great concern due to the severely limited therapeutic options and increased risk of treatment failure in patients infected with such strains (14). Our results showed that ESBL positive isolates were significantly more resistant to all tested antibiotics except for amoxiclav and imipenem compared to the non-ESBL isolates (P < 0.05). This could be due to the presence of plasmids which frequently carry both ESBL and other antibiotic resistance genes. In addition, many members of the *Enterobacteriaceae,* carry chromosomal resistance to quinolones and are commonly multidrug resistant (15). 

ESBL harboring *K. pneumoniae *isolates have been found to be resistant to other antibiotics, in particular, fluroquinolones (16-20). In studies conducted by Lautenbach in USA (2001), Shahcheraghi (2007) in Iran and Tumbarello in Italy (2006), 60%, 48% and 32% of ESBL producing isolates of *K. pneumoniae *were resistant to ciprofloxacin, respectively (16-18). A report from India also showed that 61% of *K. pneumoniae *ESBL producers were resistant to ciprofloxacin and 52% to levofloxacin (19). Resistance to ciprofloxacin has also been observed in ESBL producing *E. coli* compared to non-ESBL isolates in Iran (10). However, Eftekhar et al did not find a relationship between ESBL production and ciprofloxacin resistance, in a limited number of *K. pneumoniae *urinary isolates (20). 

In the present research, we found that resistance to all tested quinolone antibiotics was significantly higher in ESBL producing *K. pneumoniae *compared to the non-ESBL isolates. However, among the quinolones, the highest rates of resistance were observed to ciprofloxacin and nalidixic acid and there was a significant association between resistance to the two antibiotics in ESBL producing isolates. Considering the fact that ESBL and quinolone resistance genes are usually carried on mobile genetic elements and could easily disseminate among the members of *Enterobacteriaceae*, the results of this study could justify the need for setting up surveillance programs in order to avoid unnecessary antibiotic therapy and control the dissemination of resistance determinants among these pathogens. 

This study showed that antibiotic resistance and MDR were significantly higher in ESBL positive isolates compared to non-ESBL strains. Coexistence of quinolone resistance with ESBLs production is a serious public health problem and requires continuous surveillance, monitoring and revision of the antibiotic use policies.
